# Purine metabolism in sprint- vs endurance-trained athletes aged 20‒90 years

**DOI:** 10.1038/s41598-019-48633-z

**Published:** 2019-08-19

**Authors:** Jacek Zieliński, Ewa M. Slominska, Magdalena Król-Zielińska, Zbigniew Krasiński, Krzysztof Kusy

**Affiliations:** 1 Department of Athletics Strength and Conditioning, Poznan University of Physical Education, ul. Królowej Jadwigi 27/39, 61-871 Poznań, Poland; 2 Department of Biochemistry, Medical University of Gdansk, ul. Dębinki 1, 80-211 Gdańsk, Poland; 3 Department of Physical Education and Lifelong Sports, Poznan University of Physical Education, ul. Królowej Jadwigi 27/39, 61-871 Poznań, Poland; 4 Department of Vascular and Endovascular Surgery, Angiology and Phlebology, Poznan University of Medical Sciences, ul. Długa 1/2, 60-848 Poznań, Poland

**Keywords:** Ageing, Diagnostic markers

## Abstract

Purine metabolism is crucial for efficient ATP resynthesis during exercise. The aim of this study was to assess the effect of lifelong exercise training on blood purine metabolites in ageing humans at rest and after exhausting exercise. Plasma concentrations of hypoxanthine (Hx), xanthine (X), uric acid (UA) and the activity of erythrocyte hypoxanthine-guanine phosphoribosyl transferase (HGPRT) were measured in 55 sprinters (SP, 20‒90 years), 91 endurance runners (ER, 20‒81 years) and 61 untrained participants (UT, 21‒69 years). SP had significantly lower levels of plasma purine metabolites and higher erythrocyte HGPRT activity than ER and UT. In all three groups, plasma purine levels (except UA in UT) significantly increased with age (1.8‒44.0% per decade). HGPRT activity increased in SP and ER (0.5‒1.0%), while it remained unchanged in UT. Hx and X concentrations increased faster with age than UA and HGPRT levels. In summary, plasma purine concentration increases with age, representing the depletion of skeletal muscle adenine nucleotide (AdN) pool. In highly-trained athletes, this disadvantageous effect is compensated by an increase in HGPRT activity, supporting the salvage pathway of the AdN pool restoration. Such a mechanism is absent in untrained individuals. Lifelong exercise, especially speed-power training, limits the age-related purine metabolism deterioration.

## Introduction

Purine metabolism is related to adenosine triphosphate (ATP) degradation during exercise and is crucial to adenine nucleotide (AdN = ATP + ADP + AMP) pool restoration. Inosine-5′-monophosphate (IMP), inosine (Ino), hypoxanthine (Hx), xanthine (X) and uric acid (UA) are exercise-related products of AdN degradation that accumulate in skeletal muscle^1,2^ or efflux into the blood, decreasing the pool of AdN precursors^3,4^. The AdN pool may be restored through the salvage pathway from Hx^5^ or *de novo* synthesis from purine bases^6^. However, the latter process is energy-consuming^7^. The only compound that can be reutilized into the AdN pool is Hx, which is reconverted by hypoxanthine-guanine phosphoribosyltransferase (HGPRT)^5,6^.

Only a few studies assessed the changes in purine metabolite levels with increasing age. An age-related increase in Hx, X and UA concentration in blood was demonstrated in populations aged 20‒80 years, however, the significance of trends was inconsistent between studies^8–10^. The age-related change in erythrocyte HGPRT activity was investigated in healthy controls aged 26‒63 years, however, no significant trend was shown^11^. One study revealed that daily physical activity suppressed the exercise-induced increase in serum Hx in the elderly (75 ± 5 years)^12^. Because those studies solely included non-trained individuals, deprived of strong exercise stimuli, the effect of chronic intense physical training load on purine metabolism in ageing individuals could not be elucidated. In addition, only resting purine concentrations were analysed. The only study including competitive athletes revealed that professional middle-aged runners aged 37‒55 years had lower Hx concentration and higher HGPRT activity than amateur runners and non-trained participants^13^.

In young individuals (athletes under 30 years of age), purine metabolism was strongly related to training status and performance and indicated as a key adaptation, enabling a more economical distribution of energy sources for ATP resynthesis during and after exercise^14^. Research on changes in purine metabolism during short-term (6‒7-week) sprint training showed a reduction in muscle AdN pool loss^15,16^, a decrease in resting and post-exercise plasma Hx concentration^15–18^ and an increase in muscle HGPRT activity, suggesting that the reduction in exercise-induced AdN loss was one of muscle adaptations^19^. Research on one-year changes in purine metabolites in highly-trained long-distance runners^20^, middle-distance runners^21^, sprinters vs triathletes^22^ and runners of different training status^13^ revealed lowest Hx concentration in the competition phase (most intense training) and highest in the transition period (lack of training). Conversely, the highest HGPRT activity was shown in the competition phase and lowest in the transition period. Furthermore, lower plasma purine concentration and higher HGPRT activity in sprint-trained than in endurance-trained athletes indicated that high-intensity speed-power training brought about purine metabolism adaptations different from those resulting from low-intensity endurance training^14^.

The main purpose of this study is to assess the effect of lifelong exercise training on purine metabolism indices at rest and after exhausting exercise in a wide age range. We compare two opposite training models: speed-power and endurance. Our previous research has revealed that both of these training models are effective for maintaining aerobic capacity, insulin sensitivity and other health-related characteristics fundamental to ageing^23–26^. Thus, we hypothesize that ageing athletes have in general lower levels of plasma purine metabolites and higher erythrocyte HGPRT activity than their untrained peers. We also expect that plasma purine concentration increases and HGPRT activity decreases with age due to deterioration in the functioning of relevant metabolic pathways. We presume that these age-related adverse changes are less pronounced in sprint-trained master athletes who are better adapted to high-intensity exercise.

## Methods

### Participants

Two hundred seven healthy men aged 20–90 years participated in the study. Fifty-five of them were speed-power or sprint-trained athletes (SP; 55 sprinters, specialised in sprint running, jumping and combined events; age range 20–90 years), 91 were endurance runners (ER; long- and middle-distance runners; 20–81 years) and 61 were untrained individuals (UT; 21–68 years). The young athletes (20–35 years) were members of the Polish national track and field team and were included in the study within a routine periodical physiological evaluation. Athletes older than 35 years were regular participants in masters European and world championships, ranked 1–10 in their age categories and were recruited on a voluntary basis during such events by the agency of national teams’ leaders, via leaflets and personal communication. All examinations were performed during the competition phase of the annual training cycle to ensure the highest possible level of training status and reduce the effect of seasonal variation in training load and performance. Information on training history (years of participation in competitive sport) and current training volume (average hours per week over the past year) was obtained from each athlete. SP and ER athletes were involved in competitive sport for 29.2 ± 19.1 years and 26.0 ± 14.5 years, respectively, from 6 to 77 years depending on age (the relationship between training history and age: r = 0.92, P < 0.001). Weekly training duration was 8.3 ± 2.8 h · week^−1^ and 9.5 ± 3.8 h · week^−1^ in the SP and ER groups, respectively, ranging from 5.0 to 18.5 h · week^−1^ depending on age (r = −0.72 and −0.76, respectively; P < 0.001). There were no significant differences in these training characteristics between athletic groups.

UT subjects were invited through announcements via local mass media to participate in the study and to undergo preventive medical examinations. They recreationally practiced popular forms of physical activity (e.g., jogging, swimming, team games) during their leisure time, but did not exceed 150 min of moderate-intensity physical activity per week, including non-sport activities like heavy housework, gardening etc.^27^.

The aim of the research, as well as testing methodology, were explained to all participants who gave their informed consent prior to the inclusion in the study. The project was approved by the Bioethics Committee of the Karol Marcinkowski Poznań University of Medical Sciences in accordance with the Helsinki Declaration. All participants were interviewed about their health status and they underwent a preliminary clinical examination focused on cardiovascular, pulmonary and musculoskeletal systems. Blood pressure, seated after a 5–10-min quite period, was measured, and a resting 12-lead electrocardiogram was performed. The inclusion criteria were: (a) no reported history of a cardiovascular/cardiopulmonary disease or other severe/chronic diseases, (b) no major orthopaedic injury or illness resulting in inability to run, (c) no medications that could affect circulatory function, (d) normal resting electrocardiogram and (e) body mass index (BMI) below 30.0 kg·m^−2^. All participants were nonsmokers, apparently in good health and physically fit. Because of voluntary participation and the criteria mentioned earlier, the UT group was probably healthier than the general population in the same age range. Also, we did not manage to recruit untrained subjects older than 70 years who would be able to undergo a progressive exercise test until exhaustion without health risk.

### Anthropometric measurements

Participants rested for at least 24 h since the last competition, training session or other heavy exertion and arrived at the laboratory in the morning after a 12-hour overnight fast. Anthropometric measurements were performed by trained personnel according to standardised procedures. Body mass (kg) and height (cm) were measured with two decimal places using a digital measuring station (Seca 285 measuring station, SECA, Hamburg, Germany). Height and weight were measured with the participants only wearing underwear and without footwear. Body mass index (BMI) was calculated as body weight (kg) divided by height squared (m^2^).

### Maximal oxygen uptake

An incremental running exercise test was conducted at the Human Movement Laboratory of the Poznan University of Physical Education (Poznań, Poland) between 8:00 a.m. and noon, 2 h after consuming a light breakfast (bread and butter, water, without coffee or tea). Participants run on a treadmill (H/P Cosmos Pulsar, Sports & Medical, Nussdorf-Traunstein, Germany) and respiratory parameters ($\dot{V}$E, $\dot{V}$O_2_,$\,\dot{V}$CO_2_) were measured continuously (breath-by-breath) using an ergospirometric device (Cortex, MetaMax 3B^TM^ R2, Cortex Biophysik, Leipzig, Germany). All data was transmitted wirelessly to a Windows-based PC, then recorded and analysed using Meta Soft Studio 5.1.0 software (Cortex Biophysik, Leipzig, Germany). Heart rate was recorded continuously using a Polar Bluetooth Smart H6 monitor (Polar Electro, Oy, Kempele, Finland). $\dot{V}$O_2_max was expressed in ml·min^−1^ (absolute values) and in ml·kg^−1^·min^−1^ (body mass-adjusted values). Maximal oxygen pulse (O_2_ Pulse_max_) was derived by dividing $\dot{V}$O_2_max by HR_max_. The whole procedure and criteria used for achieving $\dot{V}$O_2_max were described elsewhere^25^.

### Blood sampling

Venous blood samples were taken from an antecubital vein at rest and 5 min after exercise. Blood samples were collected into two separate tubes: EDTA (2.7 ml) and lithium heparinate (4.9 ml) as an anticoagulant (S-monovette, Sarstedt, Nümbrecht, Germany). The first tube was used for the determination of haemoglobin (Hb) concentration and haematocrit (HCT). The second one was used to assess lactate (LA) in the whole blood (before separation the erythrocytes from plasma), purine metabolites in plasma and red blood cell HGPRT activity. Blood samples were centrifuged (Universal 320 R, Hettich Lab Technology, Tuttlingen, Germany) within 3 min after drawing a sample (5000 rpm, 5 min, 37 °C). The obtained plasma was deproteinised with 1.3 mol·l^−1^ perchloric acid (HClO_4_) and centrifuged (5000 rpm, 5 min, 37 °C). An acid supernatant was neutralised with 1 mmol·l^−1^ potassium carbonate (K_2_CO_3_), centrifuged (5000 rpm, 5 min, 37 °C) and stored at −80 °C before analysis. Red blood cells were bathed three times (centrifuged at 5000 rpm, 5 min, 37 °C) in an isotonic salt solution (0.9% NaCl) and then haemolysed with a hypotonic solution of Tris buffer (hydroxymethylaminomethane; 10 mmol·l^−1^, pH 7.4). The prepared haemolysate was stored at −80 °C before HGPRT activity assessment.

### Haemoglobin and lactate

Haemoglobin concentration and haematocrit value were measured using an 18-parameter automated haematology analyser (Mythic^®^ 18, Orphée, Geneva, Switzerland). The analyser aspirated 10 µL of blood during a single measurement. Lactate (LA) in whole blood (20 μl) was immediately assayed using the spectrophotometric enzymatic method (Biosen C-line, EKF Diagnostics, Barleben, Germany).

### Plasma purine metabolites: Hx, X, UA

The concentration of purine metabolites was determined in neutralised plasma using high-performance liquid chromatography (HPLC) with UV-VIS detection (Merck-Hitachi/Agilent, Japan/USA) according to the method previously described by Smoleński *et al*.^28^. The system included high-pressure gradient pump L-6200, 1050 diode array detector and autosampler AS 2000A with a thermostatic cooler set at 4 °C. Separations were carried out on the analytical column BDS Hypersil C18,150 mm × 4.6 mm × 3 μm (Thermo Finnigan, USA) protected with guard column 20 mm × 4 mm (Phenomenex, USA). All peaks were integrated and quantitative analysis was conducted using the ChemStation data system (Agilent, USA) operating on a PC. Substances were identified by comparing retention times with standards of UA, Hx and X. Quantitative analysis was conducted on a signal recorded at 254 nm for Hx and X and 280 nm for UA by comparison with external standards. The within-run/between-run %CVs were 3.1/4.1, 3.3/4.4 and 2.7/3.2% for Hx, X and UA, respectively.

### HGPRT activity

The activity of HGPRT at rest was determined using HPLC by analysis of IMP production in erythrocyte lysate. The incubation was carried out in a solution containing 125 mmol·l^−1^ Tris pH 7.4, 2.5 mmol·l^−1^ phosphoribosyl pyrophosphate (PRPP), 5 mmol·l^−1^ Hx and 13 mmol·l^−1^ MgCl_2_. The reaction was started by addition of haemolysate (25‒225 µl) and stopped with 250 µl of 0.8 mol·l^−1^ HClO_4_. It was stopped immediately in blank or incubated for 15 min and then stopped with 250 µl of 0.8 mol·l^−1^ HClO_4_. Acid extracts were neutralised with 3 mol·l^−1^ K_3_PO_4_ and then used for IMP concentration analysis by HPLC. The chromatographic system we used was described in the previous section. The mobile phase consisted of 100 mmol·l^−1^ KH_2_PO_4_ with the addition of tetrabutylammonium sulphate at a final concentration of 5 mmol·l^−1^ and pH 3.1. The separation was conducted at a flow rate of 1.2 ml·min^−1^. Retention times were ~2.0 min for Hx and 5.0 min for IMP. HGPRT activity was expressed as the amount of the produced IMP (nmol IMP∙mg Hb^−1^∙h^−1^). Haemoglobin concentrations in the erythrocyte lysates were determined by Drabkin’s method. The within-run/between-run %CVs were 3.2/4.6 for IMP.

### Statistics

There is a lack of studies showing correlations between age and purine metabolites in highly-trained older individuals. Therefore, the required sample size was estimated based on the assumption that the effect size for the relationship between age and purine metabolites levels (linear regression analysis) would range between at least small (r^2^ = 0.20) and large (r^2^ = 0.8) effect^29^. Using an α-level of 0.05 and a statistical power (1 - β) of 0.80, it was calculated that total sample size ranging from 6 to 36 participants would be needed to detect significant correlation (G*Power, Heinrich-Heine-Universität Düsseldorf, Düsseldorf, Germany). Descriptive data were expressed as mean values and standard deviations. Comparisons between the three groups of subjects were made using one-way ANOVA and post-hoc Scheffe tests when indicated by a significant *P*-value, with Shapiro–Wilk and Levene’s tests to assess the normal distribution of a sample and equality of variances, respectively. The relationships between age and purine metabolites and erythrocyte HGPRT activity were obtained by linear regression analysis. A test for parallelism of regression lines was used to determine differences between slopes. The absolute and relative cross-sectional rates of change of dependent variables with age were derived from regression equations (slopes of regression lines). Although second-order polynomial functions yielded a somewhat better fit in regression equations for some cases (*r*^2^ increase by 2–4%), the linear model was utilised due to its simplicity and clarity, without loss of essential information. The post-hoc statistical power of all regression analyses and of the most other statistics ranged from 0.80 to 1.00. Only a few isolated ANOVA analyses showed statistical power below 0.80 (0.68–0.80). All statistics were performed by using Statistica 13.0 software package (StatSoft, Inc., Tulsa, Oklahoma, USA).

## Results

Descriptive characteristics were shown in Table 1. The three groups of subjects did not significantly differ as regards the mean age and post-exercise lactate concentration. Due to training specificity, physical activity or training levels and discipline-related predispositions, there were significant between-group differences in measured somatic and physiological characteristics. The ER group had the lowest weight, height and BMI, whereas SP and UT groups were similar in this respect. The UT group had higher HR_max_ than both SP and ER groups. Absolute and relative aerobic capacity ($\dot{V}$O_2_max) was significantly higher in the ER group than in the SP group. Both athletic groups had significantly higher $\dot{V}$O_2_max than the UT group. Hb and LA_rest_ levels in the UT group were lower than in the ER group but not different from the SP group.

**Table 1:** Descriptive and exercise characteristics depending on the training profile.

	Speed-power (n = 55)	Endurance (n = 91)	Untrained (n = 61)	ANOVA *P* -value^*^	Effect size (*η*^2^)
Age (years)	47.5 ± 20.0	45.1 ± 15.7	45.1 ± 14.2	0.667	0.004
Weight (kg)	76.6 ± 8.6^‡^	71.9 ± 7.3^†#^	76.1 ± 6.4^‡^	<0.001	0.08
Height (cm)	179.1 ± 8.3	177.0 ± 5.6^`^	179.7 ± 5.0^‡^	0.025	0.04
BMI (kg·m^−2^)	23.8 ± 1.7^‡^	22.9 ± 1.9^#^	23.6 ± 1.5	0.007	0.05
HR_max_ (beat·min^−1^)	178.1 ± 11.2^†^	178.8 ± 12.8^†^	183.6 ± 9.2^‡,#^	0.015	0.05
$\dot{V}$O_2max_ (ml·min^−1^)	3583 ± 661^†.‡^	4218 ± 629^†,#^	3150 ± 534^‡,#^	<0.001	0.36
$\dot{V}$O_2max_ (ml·kg^−1^·min^−1^)	47.3 ± 8.1^†.‡^	59.1 ± 8.5^†.#^	41.6 ± 5.7^‡,#^	<0.001	0.50
Hb_rest_ (g·dl^−1^)	15.1 ± 0.9	15.3 ± 0.9^†^	14.8 ± 0.8^‡^	0.012	0.04
LA_rest_ (mmol·l^−1^)	1.1 ± 0.3	1.2 ± 0.4^†^	1.0 ± 0.3^‡^	0.002	0.07
LA_post_ (mmol·l^−1^)	9.3 ± 1.6	8.8 ± 1.9	8.7 ± 1.7	0.199	0.02
LA_post-rest_ (mmol·l^−1^)	8.1 ± 1.6	7.6 ± 1.8	7.7 ± 1.7	0.196	0.02

Data are means ± SD; ^*^between-group differences as estimated by ANOVA; post-hoc Scheffè test at *P* < 0.05: ^†^significantly different from untrained subjects. ^‡^ – significantly different from endurance-trained athletes. ^#^Significantly different from speed-power-trained athletes. *Legend:* BMI ‒ body mass index, HR_max_ ‒ maximal heart rate, $\dot{V}$O_2max_. maximal oxygen uptake; Hb ‒ haemoglobin, LA ‒ plasma lactate concentration, _rest_ ‒ resting value, _post_ ‒ post-exercise value, _post-rest_ ‒ the difference between post-exercise and resting value.

The mean level of most purine metabolites was significantly different between the groups (Table 2). Resting and post-exercise Hx concentration was lowest in the SP group, higher in the ER group and highest in the UT group. The X concentration was only significantly different between the SP and UT groups. Post-exercise UA concentration was significantly lower in the SP group than in the ER and UT groups. Resting UA concentration was only significantly different between the SP and UT groups. HGPRT activity was significantly different between all groups. The highest activity was observed in the SP group, lower in the ER group and lowest in the UT group.

**Table 2:** Plasma concentration of purine metabolites and erythrocyte HGPRT activity depending on the training profile.

	Speed-power (n = 55)	Endurance (n = 91)	Untrained (n = 61)	ANOVA *P*-value^*^	Effect size (*η*^2^)
Hx_rest_ (µmol·l^−1^)	1.7 ± 0.7^†.‡^	2.2 ± 0.5^†,#^	3.0 ± 0.9^‡,#^	<0.001	0.37
Hx_post_ (µmol·l^−1^)	17.6 ± 7.4^†.‡^	20.8 ± 5.0^†,#^	24.9 ± 5.5^‡,#^	<0.001	0.20
Hx_post-rest_ (µmol·l^−1^)	15.9 ± 6.8^†^	18.6 ± 4.6^†^	21.9 ± 4.7^‡,#^	<0.001	0.17
X_rest_ (µmol·l^−1^)	1.4 ± 0.8^†^	1.4 ± 0.6	1.7 ± 0.7^#^	0.011	0.04
X_post_ (µmol·l^−1^)	2.5 ± 0.9^†^	2.7 ± 0.7	2.9 ± 0.7^#^	0.003	0.06
X_post-rest_ (µmol·l^−1^)	1.1 ± 0.3	1.2 ± 0.4	1.2 ± 0.3	0.103	0.02
UA_rest_ (µmol·l^−1^)	299.9 ± 28.5^†^	307.8 ± 25.5	314.3 ± 27.6^#^	0.017	0.04
UA_post_ (µmol·l^−1^)	364.2 ± 42.6^†,‡^	389.2 ± 31.4^†,#^	408.2 ± 30.7^‡,#^	<0.001	0.18
UA_post-rest_ (µmol·l^−1^)	64.3 ± 21.2^†,‡^	81.3 ± 15.8^†,#^	93.8 ± 16.3^‡,#^	<0.001	0.29
HGPRT (nmolIMP ·mgHB^−1^·h^−1^)	79.1 ± 1.6^†,‡^	76.3 ± 2.1^†,#^	73.0 ± 1.7^‡,#^	<0.001	0.60

Data are means ± SD; ^*^between-group differences as estimated by ANOVA. Post-hoc Scheffè test at *P* < 0.05: ^†^significantly different from untrained subjects. ^‡^ – significantly different from endurance-trained athletes. ^#^Significantly different from speed-power-trained athletes. *Legend:* Hx ‒ hypoxanthine, HGPRT ‒ hypoxanthine-guanine phosphoribosyltransferase, UA ‒ uric acid, X ‒ xanthine, _rest_ ‒ resting value, _post_ ‒ post-exercise value, _post-rest_ ‒ the difference between post-exercise and resting value.

**Figure 1:**
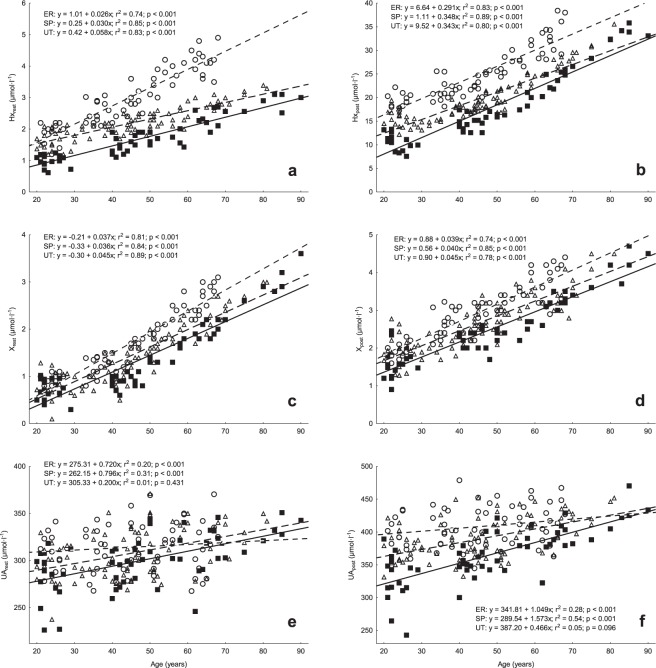
Relationships between age and plasma concentrations of purine metabolites at rest and 5 min after exercise until exhaustion in speed-power trained athletes (■, —), endurance-trained athletes (Δ, – – –) and untrained individuals (○,^……^). Resting and post-exercise hypoxanthine (panels A and B, respectively) and xanthine (panels C and D, respectively) concentrations significantly increase with age in all groups whereas uric acid (panels E and F, respectively) only increases in athletic groups.

**Figure 2:**
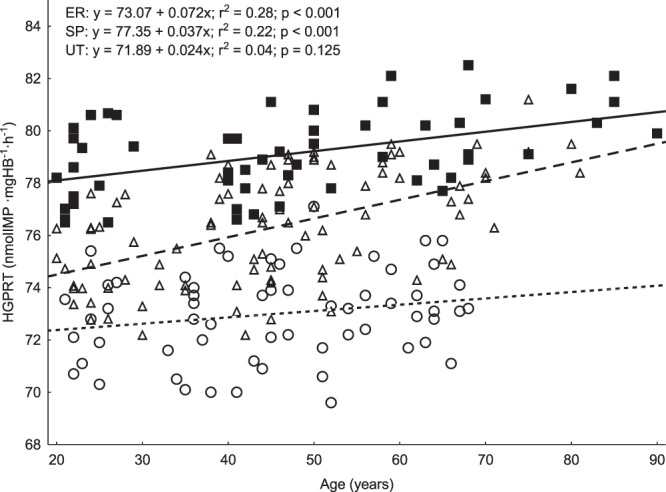
Relationship between age and red blood cell HGPRT activity at rest in speed-power trained athletes (■, —), endurance-trained athletes (Δ, – – –) and untrained individuals (○,^……^). A significant increase in HGPRT activity with age is visible in both athletic groups but not in untrained individuals.

In Fig. 1, linear relationships were shown between age and resting and post-exercise concentrations of purine metabolites. In all three groups, almost all trends were increasing and significant. The coefficients of determination (r^2^) ranged from 0.74 to 0.89 for Hx, from 0.74 to 0.89 for X and from 0.20 to 0.54 for UA (P < 0.001). The only exception was UA concentration in the UT group that was not related to age (r^2^ = 0.01‒0.05, P = 0.096‒0.431). Similarly, resting HGPRT activity was significantly related to age in the SP (r^2^ = 0.22, P < 0.001) and ER (r^2^ = 0.28, P < 0.001) groups, but not in the UT group (r^2^ = 0.04, P = 0.125) (Fig. 2).

The rate of age-related increase in purine metabolites concentration was shown in Table 3. The most pronounced significant between-group differences were observed in resting Hx concentration (*P* < 0.001, *η*^2^ = 0.33), with the highest rate in the UT group. For the rest of metabolites, the differences were trivial (*η*^2^ = 0.01‒0.06), although they reached the level of significance (*P* < 0.05) in most cases. When considering the relative change with age (percent per decade), the highest increase was observed for Hx and X (up to 44% and 37.3% per decade respectively), whereas UA concentration and HGPRT activity increased at a much slower rate (0.6‒18.2% and 0.3‒1.0% per decade, respectively) regardless of studied group.

**Table 3:** Absolute (units of measure per year) and relative (percentage per decade) cross-sectional rates of change in plasma concentration of purine metabolites and erythrocyte HGPRT activity in speed-power-trained, endurance-trained and untrained subjects.

	Speed-Power		Endurance		Untrained		Differences between slopes^*^	
	Absolute	*Relative*	Absolute	*Relative*	Absolute	*Relative*	*P*-level	Effect size (*η*^2^)
Hx_rest_ (µmol·l^−1^)	0.030^†,‡^	17.6	0.026^†,#^	17.0	0.058^‡,#^	36.7	<0.001	0.33
Hx_post_ (µmol·l^−1^)	0.347^†,‡^	43.1	0.291^†,#^	21.4	0.342^‡,#^	24.0	0.026	0.04
Hx_post-rest_ (µmol·l^−1^)	0.317	44.0	0.265	24.3	0.284	19.2	0.055	0.03
X_rest_ (µmol·l^−1^)	0.035^†^	35.0	0.036^†^	39.1	0.044^‡,#^	37.3	0.009	0.05
X_post_ (µmol·l^−1^)	0.039	29.1	0.039	23.6	0.045	25.0	0.301	0.01
X_post-rest_ (µmol·l^−1^)	0.004	4.1	0.002	1.8	0.006	7.1	0.617	<0.01
UA_rest_ (µmol·l^−1^)	0.796	2.8	0.720	2.4	0.199	0.6	0.087	0.02
UA_post_ (µmol·l^−1^)	1.573^†,‡^	4.9	1.049^†,#^	2.9	0.466^‡,#^	1.2	0.002	0.06
UA_post-rest_ (µmol·l^−1^)	0.777^†,‡^	18.2	0.329^†,#^	4.5	0.266^‡,#^	3.0	0.002	0.06
HGPRT (nmolIMP ·mgHB^−1^·h^−1^)	0.037^†,‡^	0.5	0.072^†,#^	1.0	0.024^‡,#^	0.3	0.025	0.04

^*^Significance of differences between the slopes of the regression lines.

Statistical significance between absolute rates of change: post-hoc Scheffè test at *P* < 0.05: ^†^significantly different from untrained subjects. ^‡^significantly different from endurance-trained athletes. ^#^Significantly different from speed-power-trained athletes. *Legend:* Hx ‒ hypoxanthine, HGPRT ‒ hypoxanthine-guanine phosphoribosyltransferase, UA ‒ uric acid, X ‒ xanthine, _rest_ ‒ resting value, _post_ ‒ post-exercise value, _post-rest_ ‒ difference between post-exercise and resting value.

## Discussion

To our best knowledge, this is the first study analysing the age-related change in purine metabolites in athletic cohorts up to 90 years of age. We confirmed our main hypothesis that, in fact, ageing athletes showed lower levels of plasma purine metabolites and higher resting erythrocyte HGPRT activity than their untrained peers. However, our second assumption proved to be only partly true. Admittedly, as expected, plasma purine levels significantly increased with age (except for relatively stable UA in untrained individuals), but, surprisingly, erythrocyte HGPRT activity did not decrease, showing a clearly increasing age-related trend in both athletic groups and remaining almost unchanged in the untrained group. Interestingly, contrary to our other presumption, the absolute rate of age-related increase in Hx and UA concentration was higher in sprint-trained than in endurance-trained athletes. Moreover, the picture of changes in specific metabolites was not uniform: the most dynamic age-related changes occurred for Hx and X and much smaller shifts were visible for UA and HGPRT. Below, we try to interpret these results.

Our study demonstrates that athletes undergoing lifelong sprint training are characterised by a lower resting and post-exercise plasma Hx and UA post-exercise concentrations than endurance-runners and untrained individuals. In addition, resting and post-exercise X and resting UA concentrations are different between speed-power athletes and untrained participants but not between endurance-trained and untrained groups. This suggests that chronic high-intensity training results in decreased purine efflux from skeletal muscle into the blood. Our results are supported by one previous study on the relationship between blood Hx concentration and daily physical activity in the elderly^12^. Such an adaptation is of practical importance for individuals subjected to repeated high-intensity interval exercise and gives an indication of “metabolic economy” related to the advantageous distribution of energy gained from ATP degradation^13,21,22^. In young highly-trained athletes, one-year sprint training leads to a considerable reduction in resting and post-exercise Hx levels and an increase in erythrocyte HGPRT activity compared to the effects of endurance training^22^. A similar picture can be seen in this study in a much wider age range. A stronger effect of sprint training on purine metabolism may be explained by more effective use of anaerobic (lactacid and non-lactacid) energy sources^21,22^. It shall be noted, that also chronic endurance training including a certain amount of anaerobic high-intensity exercise brings about a decrease in plasma purine concentration^13,20,22^, whereas endurance training only containing low-intensity aerobic exercise does not^13^. Thus, it can be assumed that many years’ sprint (speed-power) training, stimulating anaerobic metabolic pathways, is responsible for much lower Hx concentration compared to endurance training and recreational activity. Moreover, because ageing endurance athletes also use high-intensity exercise to a certain extent, they have lower plasma purine accumulation than untrained participants. However, in spite of the advantageous training adaptation, reflected in reduced muscle purine efflux, even in competitive athletes an increase in Hx and X is observed with age, similarly as in untrained ageing populations^8–10^. This may be interpreted as age-related worsening of the IMP-reamination pathway or increasing muscle purine production^15,19^.

The differences in Hx levels and in the rate of change between athletic groups and untrained subjects may be explained in three ways. First, sprint training results in a decrease in purine efflux into the blood after high-intensity exercise, limiting the need for energy-consuming *de novo* synthesis^15,16^. Lower plasma purine accumulation is, in turn, related to the change in the activity of some muscle enzymes, i.e. phosphofructokinase, HGPRT, AMP deaminase and nucleotide phosphorylase^19^. Second, lower plasma Hx levels reflect lower ATP and AdN muscle content after sprint training^15,19^, which is probably the case for our sprint-trained participants. Third, muscle fibre types differ in the rate of ATP degradation^30^. We assume that muscle fibre-related factors may also contribute to the differences in purine levels. In addition, it is suggested that in untrained individuals an increased cellular nucleic acid turn-over, probably caused by diseases related to ageing, e.g. arthritis or cell necrosis, is of importance^10^.

All groups investigated in this study significantly differ in erythrocyte HGPRT activity, which is highest in sprint-trained athletes and lowest in untrained participants. A similar picture is seen in highly-trained young^20–22^ and middle-aged (both elite and recreational) athletes^13^. The differences between athletes and untrained individuals can be interpreted based on *in vitro* studies showing that Hx extraction and IMP accumulation increases in human erythrocyte at decreased pH, high concentration of free phosphates and low PO_2_^31^. Such conditions accompany high-intensity exercise. This view supports our finding that HGPRT activity is higher in individuals exposed to long-term high-intensity exercise, i.e. sprint-trained athletes.

At rest, HGPRT is capable of restoring about 75% intramuscular Hx^32^. According to Hellsten-Westing *et al*.^17^, muscle resting HGPRT increases and Hx efflux into the blood decreases after a training period. So far, factors affecting skeletal muscle HGPRT activity has not been fully understood. It is suggested that purine salvage regulation depends on PRPP and ribose 5′-phosphate levels^33^. Given that the erythrocyte model is equivalent to the muscle model as regards HGPRT activity, we may speculate that in our sprinters the rephosphorylation of Hx to IMP, caused by intramuscular HGPRT activity, is more intensified than in endurance athletes. This may reflect advantageous muscle adaptation to metabolic stress, resulting in the use of the salvage pathway for AdN restoration to a greater extent than the energy-consuming *de novo* pathway. Many years’ sprint training is supposed to develop more economic pathways of purine salvage than endurance training does. However, such inference is limited because it is based on erythrocyte HGPRT, even if some analogies can be drawn (see limitation section).

Surprisingly, HGPRT activity increases with age in our master athletes, paradoxically suggesting that the metabolic mechanism behind purine salvage improves in older physically active individuals, rather than deteriorates. In healthy controls in this and another study^11^, no significant age-related HGPRT trend is observed. It seems that our findings reflect a compensatory mechanism aimed at limiting the increasing Hx production with ageing in athletic cohorts. HGPRT activity increases in response to the progressively increasing muscle Hx release into the blood. However, this mechanism is ultimately ineffective, even in competitive athletes, because the age-related rate of increase in HGPRT activity (0.3‒1.0% per decade) is by far exceeded by the rate of increase in plasma Hx concentration (18‒44% per decade). It must be noted that, even though neither athletes nor healthy controls are capable of entirely reduce this disproportion, the gap between Hx release and HGPRT activity becomes much larger in untrained individuals (unchanging HGPRT activity and fastest Hx increase with age), suggesting a positive effect of sport training on preservation of normal purine metabolism, especially on IMP salvage and AdN pool restoration. Among athletes, sprint-trained individuals seem to show more effective mechanisms of purine salvage (lower Hx levels and higher HGPRT activity across the whole age range) than endurance-trained ones, in spite of blurred differences in oldest age categories (~90 years of age). To our best knowledge, there is only one previous study, by Stolk *et al*.^11^, dealing with age-related change in HGPRT activity in a small group of healthy controls aged 26‒63 years. In support of our results, they do not show any significant trend in HGPRT activity (~0.09 unit per year) in untrained participants, confirming the view that only vigorous physical activity can delay the purine metabolism degradation.

HGPRT activity and its age-related change is indirectly associated with the purine nucleotide cycle (PNC), which is another stage of the metabolic pathways related to ATP degradation and resynthesis. IMP molecules, formed via AMP deamination, *de novo* synthesis, or salvage pathway using HGPRT, are then reaminated to AMP within the PNC via reactions catalysed by adenylosuccinate synthase and adenylosuccinase, to restore the AdN pool. It is suggested that the age-related decrease in IMP concentration is due to a slowdown in IMP synthesis^34^. Most likely, in untrained ageing individuals, the compensatory mechanism (salvage via HGPRT), supplying IMP to the PNC cycle, becomes less or non-efficient, which is reflected by a higher level of plasma purine metabolites and lower HGPRT activity. Our study suggests that the Hx salvage using HGPRT is critical in aged people and that chronic exercise, especially of short-duration and high-intensity character (speed-power), supports ATP restoration and limits purine metabolites production at rest and post-exercise.

We have not found significant differences between SP and ER groups in resting UA but there are clear differences as regards post-exercise values. It is consistent with our earlier study on young sprinters and triathletes^22^. Increased UA production primarily begins after Hx release from exercising skeletal muscle into the blood and Hx is further intensively degraded to UA in the liver^35^. Therefore, post-exercise UA levels in athletes reflect the degradation of its main precursor (i.e. Hx), described in the earlier section. We have also revealed that plasma resting and post-exercise UA concentration increases with age in athletes but not in untrained individuals. In support of our results, blood UA concentration does not undergo any significant age-related change also in other healthy ageing population, in spite of the simultaneous increase in Hx and X levels^10^. However, the picture of age-related UA concentration change is ambiguous. In a study by Culleton *et al*.^8^, serum UA levels increase with age (20‒80 years) in women but not in men. Kuzuya *et al*.^9^ suggest that serum UA levels increase with advancing age in both men and women. Irrespective of the data obtained from untrained populations, it seems that long-term physical training brings about a clear age-related increase in plasma UA. However, it must be noted that young athletes start from a considerably lower UA levels (Fig. 1e,f) than untrained participants. Only in older individuals (~70‒90 years of age), the differences disappear to a great extent. Thus, the discrepancies between athletes and non-athletes in the rate of age-related increase in UA concentration mainly result from differences in initial values which are lower in trained individuals. Probably, ageing processes even out the differences.

Our data suggest that simultaneous age-related increases in plasma Hx concentration and erythrocyte HGPRT activity reflect the degradation of metabolic function resulting in increased muscle AdN pool degradation and its delayed restoration via energy-consuming *de novo* synthesis. Consequently, an increased accumulation of plasma purine metabolites and a decrease in exercise economy are observed. Even the compensatory intensification of HGPRT activity does not keep pace with the age-related increase in plasma purine derivatives concentration, especially Hx. Therefore, in ageing people adequately long recovery periods are required after high-intensity exercise (session) to restore AdN pool, because the slow and energy-consuming *de novo* pathway rather than the quick salvage pathway is used^2^. Exercise training slows down this detrimental process as shown in our study and accelerates post-exercise recovery as demonstrated in research on young participants undergoing sprint training^15,16,36^. However, even as much as 120 min may be insufficient to decrease plasma Hx to pre-exercise levels^36^.

Purine metabolism deterioration with age may be also seen in a wider perspective. Physical training can, and indeed should, substantially support metabolic diseases treatment and prevention ‒ two key problems in contemporary medicine and health sciences ‒ even though optimal exercise load (intensity, duration, frequency etc.) is still under discussion. Regardless of debatable details, through which exercise affects metabolic mechanisms behind the lifestyle- and ageing-related disorders, exercise proves to be effective, because it most often activates and strengthens relevant metabolic mechanisms and adaptations more effectively than drugs^24,37,38^. This also refers to diseases consisting in purine pathways impairment (e.g. hyperuricaemia) where even relatively short periods of light aerobic exercise, contrary to drugs, result in a decrease of serum uric acid levels by accelerating ATP turnover rate^38^. In our study, in both highly-trained and recreationally active participants, resting UA concentration does not exceed the upper normal limit across the whole age range (20‒90 years), showing a negligible risk of hypeuricaemia. In addition, which is not considered by other researchers^38^, plasma purine response to exercise is attenuated in trained people, i.e. the net post-exercise increase in Hx and UA concentrations is lower than in less trained subjects (Table 2), which reflects a more economical purine metabolism in physically active people.

Our study has some limitations. Physical fitness and sport performance of our participants are above the average of the general population. They usually start their intensive training in childhood and continue it over years. They are a “model population” of successful ageing, less exposed to health risk factors typical for older age (inactivity, diseases of affluence etc.). Also, genetic factors related to preselection for a particular sports discipline may influence the results. Thus, presented views and recommendations may not be uncritically transferred to sedentary individuals or patients suffering from specific ailments who are able to tolerate a smaller portion of exercise than healthy highly-trained ones. Moreover, only a cross-sectional analysis is presented, showing approximate trends in analysed variables. Nevertheless, the obtained picture seems to be valuable, because longitudinal data in such a wide age range are difficult to attain. A potential limitation of our study is blood sampling at the 5th min of the recovery period. Since Hx concentration peaks 10‒20 min post-exercise^3,39^ and UA concentration even later, up to 30 min^15,16^, samples obtained later would provide additional information. However, early sampling makes it possible to successfully detect between-group differences and is more practical in the context of exercise diagnostics^13,20,21,23,40^. We could not perform a muscle biopsy due to legal regulations. We are aware that, since the relationship between muscle and erythrocyte metabolism is unclear, erythrocyte HGPRT activity may be considered debatable. Unfortunately, erythrocytes do not contain enzymes that transform IMP into AMP^41^, thus do not have *de novo* capacity^19^. However, we assume that erythrocyte activity can serve as a simple and available model aptly reflecting overall metabolic changes in skeletal muscle. The strengths of our study are: (i) a wide age range of participants, so far unprecedented in research on purine metabolism and HGPRT activity, (ii) highly-trained athletes clustered in well-defined uniform groups representing distinct training models, (iii) all measurements performed in the competition period to avoid the effect of seasonal changes, (iv) a relatively large group of speed-power athletes performing an endurance test, (v) purine metabolites measured for the first time in older participants after a progressive exercise until exhaustion and (vi) inclusion of the control group.

In conclusion, we demonstrate that ageing athletes (20‒90 years) show lower levels of plasma purine metabolites and higher erythrocyte HGPRT activity than their untrained peers. Chronic sprint training brings about lower levels of plasma purine metabolites and higher erythrocyte HGPRT activity than endurance training and recreational activity do. In highly trained athletes, plasma purine concentration increases with age, representing the loss of the skeletal muscle AdN pool. This disadvantageous change is, however, accompanied by a compensation mechanism including significant HGPRT activity increase, supporting the salvage pathway of the AdN pool restoration. Such a mechanism is absent in untrained individuals in which, admittedly, an increase in purine concentration is also observed but, contrary to athletes, no significant change in HGPRT activity is visible. The results of this study suggest that lifelong physical training, in particular based on speed-power exercise, considerably limits purine metabolism deterioration.

## Data Availability

The datasets analysed during the current study are available from the corresponding author on reasonable request.
